# Large Language Models in equity markets: applications, techniques, and insights

**DOI:** 10.3389/frai.2025.1608365

**Published:** 2025-08-27

**Authors:** Aakanksha Jadhav, Vishal Mirza

**Affiliations:** Independent Researcher, New York, NY, United States

**Keywords:** Large Language Models, LLMS, finance, NLP, stock, equity, investing, algorithmic trading

## Abstract

Recent breakthroughs in Large Language Models (LLMs) have the potential to disrupt equity investing by enabling sophisticated data analysis, market prediction, and automated trading. This paper presents a comprehensive review of 84 research studies conducted between 2022 and early 2025, synthesizing the state of LLM applications in stock investing. We provide a dual-layered categorization: first, by financial applications such as stock price forecasting, sentiment analysis, portfolio management, and algorithmic trading; second, by technical methodologies, including prompting, fine-tuning, multi-agent frameworks, reinforcement learning, and custom architectures. Additionally, we consolidate findings on the datasets used, ranging from financial statements to multimodal data (news, market trends, earnings transcripts, social media), and systematically compare general-purpose vs. finance-specialized LLMs used in research. Our analysis identifies key research trends, commonalities, and divergences across studies, evaluating both their empirical contributions and methodological innovations. We highlight the strengths of existing research, such as improved sentiment extraction and the use of reinforcement learning to factor market feedback, alongside critical gaps in scalability, interpretability, and real-world validation. Finally, we propose directions for future research, emphasizing hybrid modeling approaches, architectures that factor reasoning and large context windows, and robust evaluation frameworks to advance AI-driven financial strategies. By mapping the intersection of LLMs and equity markets, this review provides a foundation and roadmap for future research and practical implementation in the financial sector.

## 1 Introduction

Historically, investment strategies largely relied on structured data, fundamental and technical analysis, and human interpretation of financial reports, often resulting in slow decision-making and limited market adaptability. The advent of Large Language Models (LLMs) marks a transformative era in equity investing, shifting the paradigm from traditional, manual analysis to automated, real-time insights. LLMs now enable the rapid processing and integration of vast datasets, combining structured financial metrics with unstructured sources like news, earnings call transcripts, and social media sentiment. This integration uncovers market trends and signals with unprecedented precision, converting previously overlooked textual data into actionable trading signals. Moreover, LLMs are facilitating the evolution from static, rule-based models to dynamic, self-learning systems powered by reinforcement learning and multi-agent frameworks. This evolution enhances market responsiveness, improves risk management, and boosts alpha generation by identifying complex market narratives and emerging shifts. However, the integration of LLMs into equity investing is not without its challenges, including data reliability, potential biases, regulatory considerations, and the interpretability of AI-driven recommendations.

This review paper analyzes current research on the application of Large Language Models (LLMs) in equity markets. It focuses on the following research questions: (1) What are the major trends in how LLMs are being applied within equity markets? (2) What are the primary technical innovations and methodological approaches employed in LLM-driven equity research? (3) What are the significant limitations, challenges, and research gaps that have been identified in the literature?

### 1.1 Key considerations for LLM usage in stock investing

#### 1.1.1 Data complexity

Multimodal data: The financial stock investing landscape is characterized by an increasingly complex and voluminous collection of multi-modal data, presenting significant analytical challenges. Investors must process both structured and unstructured data sets, each requiring distinct computational approaches. Structured data, including financial statements, earnings reports, and quantitative market metrics demands rigorous statistical and analytical modeling. In contrast, unstructured data, such as financial news, social media sentiment, and analyst reports, necessitates advanced natural language processing (NLP) techniques to extract meaningful insights. Additionally, the integration of visual elements such as price charts, technical indicators, and graphical financial summaries, along with audio or text data from earnings calls and investor briefings, further complicates the analytical landscape.

Large datasets: The size of datasets used to analyze a single stock, or a portfolio is large and complex with inclusion of multimodal data- multiple unstructured and structured datasets and real-time data streams. Additionally, the size of financial earning reports (quarterly, annual, 10Q, 10 K) and industry report pdf files usually spans 100+ pages, while earning transcripts contains large audio/text files.

Why LLMs can address data complexity in large datasets: Large Language Models (LLMs) are uniquely suited to handle the data complexity inherent in financial stock investing due to their ability to process and synthesize large volumes of heterogeneous data. Unlike traditional models that require separate preprocessing pipelines for each data type, LLMs are trained on vast corpora of multimodal information, enabling them to natively handle unstructured data such as text from earnings reports, analyst notes, and news articles. Their contextual understanding allows them to extract relevant insights from lengthy documents (e.g., 100+ page 10-Ks), and their ability to summarize, infer sentiment, and answer questions from natural language inputs makes them ideal for navigating and distilling large, complex datasets. Moreover, LLMs can be extended or paired with vision and speech models (e.g., via multimodal architectures like GPT-4V or Gemini) to interpret visual data (charts, tables) and audio transcripts (earnings calls), thereby providing a unified framework for holistic financial analysis.

#### 1.1.2 Time sensitivity and real time analysis

Financial markets are inherently time-sensitive, with investment decisions often hinging on the rapid processing and analysis of information. Latency and response times play a pivotal role, particularly in short-term trading strategies such as day trading. The ability to react swiftly to market fluctuations can significantly impact investment outcomes, emphasizing the need for real-time or near-real-time analytical capabilities. This consideration is especially pertinent when evaluating the suitability of Large Language Models (LLMs) for equity investing, as their effectiveness depends on their ability to process vast amounts of financial data with minimal delay.

#### 1.1.3 Diverse investment strategies and asset classes

Investment strategies in equity markets vary widely, from long-term value and growth investing to short-term momentum and high-frequency trading, each requiring distinct analytical methods. Additionally, this complexity extends across asset classes, including stocks, ETFs, and derivatives like options and futures, each with unique characteristics and risk factors. While LLMs hold promise for financial investing, effectively adapting them to diverse asset classes and investment styles remains a critical challenge. Additionally, financial analysis encompasses a wide range of approaches, including fundamental and technical analysis. Fundamental analysis focuses on the intrinsic value of assets, while technical analysis examines historical price and volume data.

### 1.2 Review scope

The application of LLMs to stock and equity investing has seen a significant surge in research, particularly in the 2 years following the late 2022 launch of ChatGPT, during which we identified approximately 84 relevant studies. Despite the growing interest in applying LLMs to finance and stock investment, the rapid proliferation of research in this domain has created a fragmented landscape. Some studies focus on multi-agent trading frameworks, others explore time series forecasting, while still others develop domain-specific LLM architectures. This review aims to consolidate these disparate efforts by synthesizing findings from 84 recent studies. Our goal is to provide a comprehensive overview of the research on how LLMs are being applied to transform stock and equity investing, while also highlighting key challenges and gaps in the field.

We selected 84 research papers from a comprehensive set of 100+ research papers sourced from Google Scholar and arXiv. The selection process involved applying keyword filters such as “LLM for Stock Investing” and “Large Language Models for Equity Investing. Papers that primarily focused on macroeconomic analysis or general financial risk modeling, broader finance topics without explicit application of LLMs to equity markets, were excluded.

To provide a holistic analysis, we adopt a two-fold classification approach:

1. Applications of LLMs in Finance-Equity Investing (Section 2): This section categorizes the practical goals (why) and real-world relevance (what) of LLM applications in equity investing.
2. LLM Technical Innovations and Approaches (Section 3): This section examines the method (how)—detailing the specific techniques and methodologies used in LLM applications.

This dual approach enables a comprehensive evaluation of both the “why, what” and “how” aspects of research on LLM usage in equity investing. By exploring the key observations and insights gained from existing research, we seek to highlight both the potential and the limitations of LLMs in this dynamic and challenging field. As investors increasingly integrate LLMs and other reinforcement learning AI techniques into their decision-making processes, understanding the potential and limitations of LLM-driven strategies becomes crucial. This paper serves as both a roadmap and a call to action for researchers and practitioners, paving the way toward more transparent, efficient, and reliable applications of LLMs in stock and equity investing. For simplicity, brevity, and readability we refer to each research paper by ‘Paper No.’ as indexed in the References section and avoid the use of complete paper title and author names unlike the format of traditional literature reviews.

## 2 Applications of LLMs in finance-equity investing

**Figure 1:**
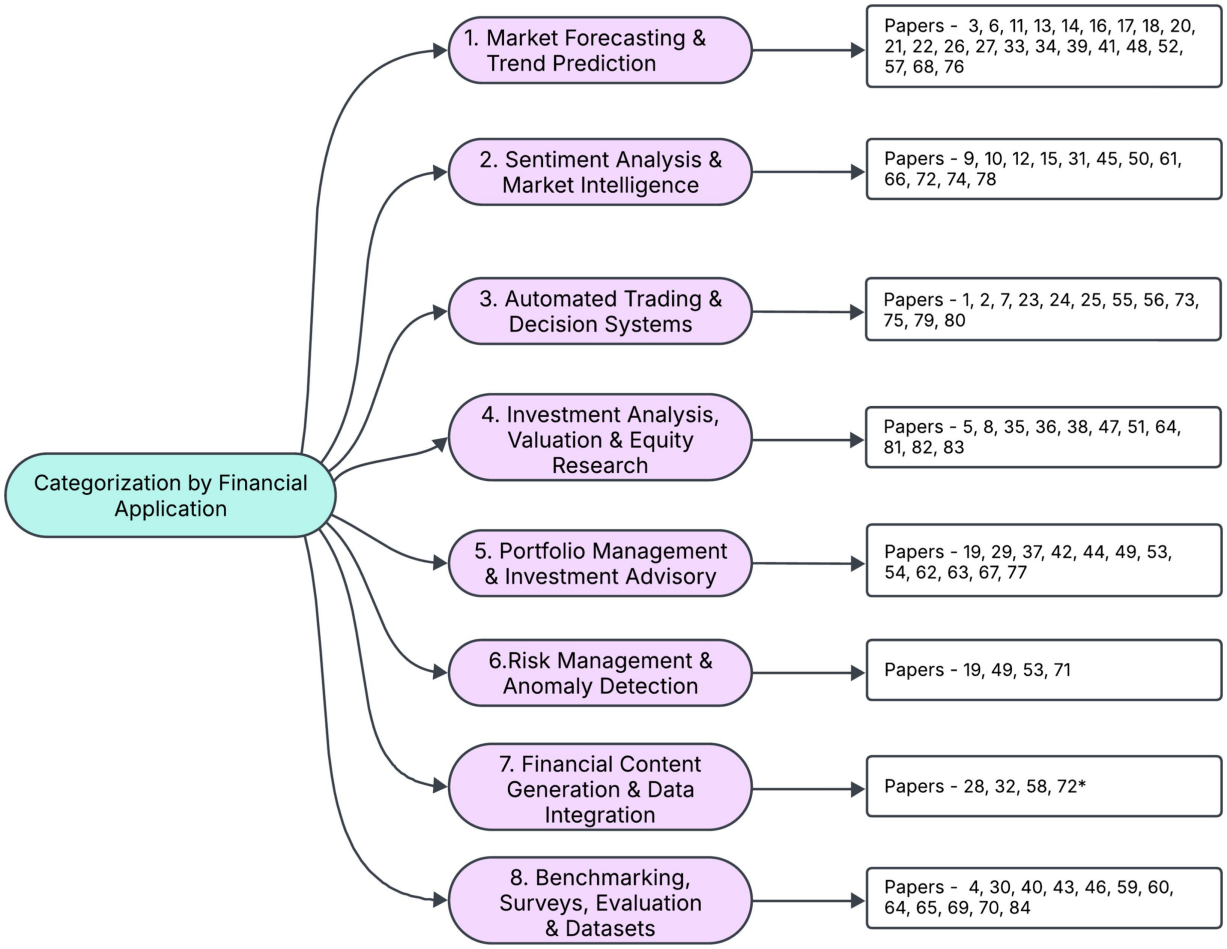
Categorization of research by financial application for stock investing.

An effective method for categorizing the research landscape of LLM applications in stock investing is by end use or financial application, as it reflects how both practitioners and researchers typically frame their objectives. By grouping the 84 papers into categories such as stock trend prediction, sentiment analysis, portfolio management, and others we highlight each study’s practical goals and real-world relevance (Figure 1). This application-centric perspective not only spotlights potential synergies among similar works but also reveals gaps in coverage—for example, relatively few studies address risk management tasks. Consequently, categorizing by end use provides a clear, pragmatic lens through which to assess the collective impact and future directions of LLM research in equity and stock investing.

### 2.1 Stock price forecasting and market trends

A substantial body of research on LLMs in equity investing centers on forecasting stock prices, predicting market returns, and analyzing broader market trends. Typically, these studies combine textual data (e.g., news, press releases, earnings reports, analyst reports, social media content, etc.) with quantitative time-series data to enhance predictive accuracy beyond traditional methods.

#### 2.1.1 Integration of qualitative data in forecasting

Leveraging language models to extract and interpret market signals from unstructured data has proven effective in complementing numerical datasets for forecasting models. This subcategory explores how LLMs integrate unstructured textual data with structured financial indicators to improve stock prediction models and generate actionable market signals.

##### 2.1.1.1 Stock prediction using multiple data sets

- Shi and Hollifield (2024) compares GPT-4 and BERT for stock return prediction using historical economic indicators, showing the strength of LLMs in processing structured economic data.
- Ni et al. (2024) forecasts post-earnings stock performance by combining textual earnings reports and transcripts with key financial metrics.
- Lee et al. (n.d.) transforms qualitative textual insights into quantitative market signals, offering a direct mapping approach for LLM-based forecasting.
- Ding et al. (2023) fuses LLMs with time-series modeling to improve stock return predictions in the Chinese market, illustrating LLM adaptability to local market contexts.
- Swamy et al. (n.d.) blends LLM-generated “priority indices” with traditional quantitative factors to enhance market trend prediction.
- Tong et al. (2024) introduces Ploutos, which integrates textual and numerical data to generate interpretable forecasts of price movements, focusing on explainability.
- Cheng and Tang (2023) demonstrates GPT-4’s ability to design high-performing investment factors with strong returns and Sharpe ratios.
- Fatemi and Hu (2024) proposes a multi-agent, multimodal system for stock prediction, emphasizing complex agent-based modeling over single-model forecasting.
- Deng et al. (2024) introduces a “denoising-then-voting” method to improve few-shot stock forecasting in noisy and data-scarce environments.
- Di et al. (2024) and Guo and Shum (2024) extend forecasting to broader market indices using LLMs combined with multi-source datasets and knowledge graphs, respectively.

##### 2.1.1.2 Stock prediction using news data sets

- Tandon (2024) compares BERT and FinBERT for general sentiment extraction from news headlines, establishing baseline model differences.
- Bhat (n.d.) focuses on extracting emotional tone from news headlines to forecast stock movements, highlighting finer-grained sentiment modeling.
- Liang et al. (2024) introduces FinGPT, which incorporates contextual nuances from financial news to improve prediction accuracy.
- Chen et al. (2022) shows that complex linguistic features captured by LLMs outperform traditional models in global stock return forecasting.
- Guo and Hauptmann (2024) fine-tunes LLMs using Mistral embeddings on financial news, demonstrating performance gains in portfolio returns from domain-specific training.
- Vidal (2024) reports mixed results with certain LLMs, cautioning against overreliance without robust evaluation.
- Lopez-Lira and Tang (2023) evaluates ChatGPT’s raw capability to predict stock prices from headlines without financial fine-tuning, benchmarking general-purpose LLMs against domain-trained ones.

These studies collectively demonstrate the value of combining LLMs with structured and unstructured data for market prediction, showcasing methodological diversity and a trend toward interpretability and domain adaptation.

#### 2.1.2 Time series specialization

This subcategory focuses on adapting LLMs to handle temporal dependencies and structured price signals for enhanced time-series forecasting in financial markets.

- Valeyre and Aboura (2024) evaluates LLMs on a dataset of major U.S. equities, demonstrating their alpha-generating potential in traditional time-series forecasting tasks.
- Wang et al. (2024) proposes a novel architecture that fuses textual inputs with time-series signals to improve predictive accuracy in stock price movements.
- Chen et al. (2024) investigates the role of historical return patterns in informing future price predictions, using LLMs to extract and model temporal patterns.
- Voigt et al. (2024) explores the methodological convergence between NLP and time-series analysis, applying LLMs to structured forecasting problems in quantitative finance.
- Wang et al. (2024) introduces Stock time—a bespoke architecture specifically designed for financial time-series prediction, optimizing LLM capabilities for sequential modeling tasks.

These studies highlight how LLMs are being repurposed or architecturally enhanced to address the unique demands of time-dependent financial data.

### 2.2 Sentiment analysis and market intelligence

Research in this domain focuses on extracting, quantifying, and interpreting sentiment from diverse textual sources—such as news articles, social media posts, analyst reports, and press releases—to generate actionable intelligence for market analysis. The key themes include.

#### 2.2.1 Text mining and natural language processing

This category focuses on extracting insights from financial text data using LLMs, with a strong emphasis on sentiment analysis across various domains and data types.

- Deng et al. (2022) applies LLMs to Reddit data to extract investor sentiment, showcasing the utility of social media as an alternative sentiment source.
- Das et al. (2024) evaluates LLMs for single-stock trading, integrating news sentiment and price movement signals to inform trading decisions.
- Wu (2024) investigates the relationship between market sentiment from news sources and resulting stock price fluctuations.
- Zhao and Welsch (2024) introduces an adaptive LLM framework for sentiment analysis, integrating instruction tuning and real-time market feedback to improve adaptability.
- Aparicio et al. (2024) presents BioFinBERT, a domain-specific LLM fine-tuned for biotechnology stock sentiment, highlighting the value of industry-specific modeling.
- Liu et al. (2024) explores the correlation between news sentiment and Bitcoin prices, extending sentiment analysis to cryptocurrency markets.
- Xing (2024) proposes a multi-agent LLM system that enhances sentiment classification accuracy through collaborative agent dynamics.
- Dolphin et al. (2024) develops an LLM-driven system to process unstructured financial news and tickers, converting them into structured data formats for sentiment tracking.
- Yu (2023) conducts a case study on US equity market news, examining the variability and reliability of LLM-generated sentiment labels.
- Kirtac and Germano (2024) compares general-purpose and fine-tuned LLMs for financial sentiment analysis, analyzing performance differences across model types.

These studies reflect a broad spectrum of sentiment analysis techniques using LLMs—from basic extraction to adaptive and agent-based frameworks—demonstrating their growing sophistication and importance in financial modeling.

#### 2.2.2 Sentiment scoring

This subcategory focuses on converting qualitative sentiment from financial text into quantitative scores that can directly inform trading and investment strategies.

- Bond et al. (2023) uses ChatGPT to generate a sentiment-based market indicator from daily news summaries, demonstrating superior performance compared to traditional sentiment analysis methods.
- Lefort et al. (2024) applies ChatGPT to financial news headlines to derive sentiment scores for NASDAQ index predictions, integrating these scores into an NLP-driven investment strategy.

Both studies illustrate how LLMs can translate textual sentiment into actionable numeric signals, with Paper 31 emphasizing broader market trends and Paper 61 focusing on NASDAQ-specific movements.

### 2.3 Automated trading and decision systems

This category highlights research dedicated to building systems capable of autonomously making trading decisions or crafting strategies based on LLM outputs. These approaches range from multi-agent frameworks to fully functional trading bots, operating in both simulated and real-world environments.

#### 2.3.1 Algorithmic trading/automated trading decision systems (AI agents)

- Yu et al. (2024) introduces a multi-agent framework FinCon designed to handle complex financial tasks, including trading and portfolio management.
- Kou et al. (2024) presents a multi-agent methodology for quantitative stock investing, combining LLMs with established quantitative techniques to enhance performance and stability.
- Zhang et al. (2024) simulates investor behavior through an LLM-driven multi-agent system (Stock Agent) that adapts to live market conditions.
- Xiao et al. (2024) deploys specialized LLM-based agents (Trading Agents) within a structure modeled on real-world trading firms, demonstrating improved performance against standard benchmarks.
- Li et al. (2023) introduces TradingGPT, a multi-agent framework with layered memories and distinct agent characters, aiming to emulate human cognitive processes for improved trading efficiency and accuracy, emphasizing the hierarchical nature of human memory.
- Yu et al. (2023), FinMem, addresses the need for a novel LLM agent architecture to effectively transition from question-answering to purpose-driven financial trading, focusing on multi-source information processing, reasoning chains, and task prioritization.
- Wang et al. (2024) investigates LLM reasoning processes for trading decisions based on trend observations in crypto trading, revealing that less sophisticated LLMs can outperform more sophisticated LLMs, offering a contrast to the trend of increasing model complexity.
- Li et al. (2024) develops an LLM-based trading agent, CryptoTrade, that integrates diverse data (on chain and off-chain data) for cryptocurrency trading, showcasing LLM versatility beyond traditional stock markets.

#### 2.3.2 Sentiment analysis for trading/portfolio management

- Konstantinidis et al. (2024) proposes a sentiment-analysis framework (FinLlama) for algorithmic trading that elevates portfolio returns, even in volatile markets.
- Chen et al. (2024) explores social media sentiment to inform trading strategies, linking shifts in investor sentiment to returns and herding behavior within AI-driven trading ecosystems. This study demonstrates the potential of factoring investor sentiment to inform trading decisions.

#### 2.3.3 Adaptive trading/reinforcement learning

- Saqur and Rudzicz (2024) introduces Reinforcement Learning from Market Feedback (RLMF), enabling LLMs to adapt continuously to evolving market dynamics.

#### 2.3.4 AI agent platforms

- Yang et al. (2024) provides an open-source AI agent platform, FinRobot, broadening access to specialized LLM-driven tools for both researchers and practitioners.

Integrating sentiment analysis (2.3.2) into algorithmic systems (2.3.1) combines quantitative and qualitative data. Reinforcement learning (2.3.3) enhances agent adaptability. Open-source platforms (2.3.4) broaden access. These advances demonstrate LLMs’ transformative potential in automated trading.

### 2.4 Investment analysis, valuation and equity research

Large Language Models (LLMs) offer considerable potential for streamlining and enhancing traditional equity research. By automating tasks such as stock ratings, identifying new investment opportunities, and assisting in the interpretation of complex financial documents, LLMs can significantly improve analysts’ efficiency and insights. Research in this domain can be grouped into several key themes.

#### 2.4.1 Equity research automation

- Papasotiriou et al. (2024) presents a method for automating and improving equity stock ratings by combining GPT-4 with multimodal financial data.
- Li et al. (2023) leverages generative AI (Llama2 and GPT-3.5) to automate fundamental investment research, with a focus on data summarization and ideation.
- Zhou et al. (2024) introduces FinRobot, an open-source AI agent designed for sell-side analysts seeking to automate equity research processes.
- Yue and Au (2023) describes GPTQuant, a conversational chatbot that simplifies investment research by generating and executing Python code.

While all papers focus on automation, they differ in their approach. (Papasotiriou et al., 2024) focuses on ratings, (Li et al., 2023) on idea generation, (Zhou et al., 2024) on open-source tools, and (Yue and Au, 2023) on user interaction, highlighting the diversity of automation strategies.

#### 2.4.2 Investment research/analysis

- Kim and Oh (n.d.) presents a novel approach that combines LLMs, NLP, and dynamic data retrieval for in-depth stock market analysis.

#### 2.4.3 Stock selection/portfolio management

- Fatouros et al. (2024) proposes MarketSenseAI, a GPT-4–based framework that supports stock selection.
- Fatouros et al. (2025) extends this approach with MarketSenseAI 2.0, an enhanced LLM-driven system that integrates various financial datasets and Retrieval-Augmented Generation to optimize portfolio performance.

Both papers focus on stock selection, but Paper 47 expands on Paper 8 by incorporating more diverse data and advanced generation techniques to improve portfolio optimization.

#### 2.4.4 Executive/corporate communication analysis

- Chiang et al. (2025) explores how LLMs can evaluate Q&A segments in earnings calls to assess the transparency and responsiveness of corporate executives, providing valuable insights for investment decision-making.

#### 2.4.5 Modeling

- Wang et al. (2023) introduces a new paradigm for alpha mining in quantitative investment, addressing the challenge of translating quant researchers’ ideas into effective trading strategies.
- Wang et al. (2024) focuses on using Large Language Models (LLMs) to help understand and model investor decision-making, especially when investors are influenced by “herd behavior” (following the crowd).

Wang et al. (2023) focuses on modeling quant researcher ideas, while (Wang et al., 2024) model’s investor behavior, showing two different modeling approaches. Building upon the modeling of financial data, researchers are also looking at how to improve the explainability of LLM models.

#### 2.4.6 Explainability/interpretability

The focus on explainability is important for the real-world application of the models.

- Koa et al. (2024) develops a self-learning framework to enhance the interpretability of stock predictions by generating human-readable explanations, addressing a critical challenge for both traditional models and LLMs.
- Additionally, Lopez-Lira and Tang (2023), Tong et al. (2024), Abdelsamie and Wang (2024), Zhao (2024) cover model interpretability. Tong et al. (2024) and Lopez-Lira and Tang (2023), introduce novel frameworks and Zhao (2024) introduces a novel algorithm for enhancing interpretability for equity application of LLMs, while (Abdelsamie and Wang, 2024) compares interpretability across general purpose LLMs.

### 2.5 Portfolio management and investment advisory

Research in this domain applies Large Language Models (LLMs) to portfolio construction, wealth management, and personalized financial advice. The overarching aim is to optimize asset allocations and provide actionable recommendations for investors. Key themes include:

#### 2.5.1 Portfolio construction/optimization

- Lu et al. (2023) demonstrates high-alpha portfolio generation by incorporating insights from news and policy announcements.
- Romanko et al. (2023) uses ChatGPT for stock selection in portfolio construction, integrating it with traditional optimization methods.
- Ko and Lee (2024) explores how ChatGPT can assist in asset class selection and enhance portfolio diversification.
- Gu et al. (2024) proposes an adaptive portfolio management framework that leverages LLMs and Reinforcement Learning for dynamic long–short positions.
- Huang et al. (2024) introduces a novel LLM-based, algorithm-driven system for stock selection and portfolio optimization.
- Zhao (2024) to revolutionize portfolio management by overcoming limitations in traditional approaches, this research develops a framework integrating advanced NLP, LLMs, and DRL (Deep Reinforcement learning) for enhanced return predictions, sentiment extraction, and optimized trading strategies.
- Perlin et al. (n.d.) evaluates Google’s Gemini 1.5 Flash LLM’s investment performance using extensive U.S. market data, finding it does not consistently outperform basic benchmarks, and its risk-adjusted returns decline with longer investment horizons. It covers a large-scale simulation of investment decisions using different data inputs and time horizons.

While (Lu et al., 2023; Romanko et al., 2023; Ko and Lee, 2024; Gu et al., 2024; Huang et al., 2024; Zhao, 2024) provide frameworks for portfolio construction, Perlin et al. (n.d.) differentiates itself by evaluating an existing and widely utilized LLM, showing the difference between theoretical implementations and real-world evaluation. Also, the complexity of frameworks increases for (Gu et al., 2024; Huang et al., 2024; Zhao, 2024) including a reinforcement learning approach. Some papers utilize hybrid approaches, while other papers rely solely on LLMs.

While the research conducted within the sphere of portfolio construction and optimization can, lead to, and improve the quality of Robo-advisory platforms, we cover that in as separate section below.

#### 2.5.2 Robo-advisory/investor education

- Fieberg et al. (2024): Illustrates how LLMs can generate financial advice tailored to individual investor profiles.
- Lu (2025): Examines the impact of varying levels of financial literacy (alpha/beta) on investor behavior within robo-advisory platforms.

### 2.6 Risk management and anomaly detection

While much of the literature focuses on improving predictive accuracy, few studies address the robustness and reliability of LLMs in equity investing. Three key themes emerge:

#### 2.6.1 Bias assessment

- Glasserman and Lin (2023) investigates two potential biases that can arise when LLMs use news sentiment for stock predictions: Look-Ahead Bias—when models inadvertently incorporate future returns into current forecasts. Distraction Effect—where extraneous company information skews the sentiment assessment.

#### 2.6.2 Anomaly detection

This category of research examines methods for identifying unusual market conditions or portfolio crashes.

- Park (2024) introduces an LLM-driven multi-agent framework designed to automate anomaly detection in financial markets, reducing the burden of manual alert validation. Similarly,
- Koa et al. (2024) proposes a framework called “Temporal Relational Reasoning (TRR),” which utilizes LLMs to detect portfolio crashes by applying human-like temporal reasoning.
- Yang et al. (2025) proposes TwinMarket, a multi-agent framework that simulates complex human behavior. Simulated stock market experiments show how individual actions lead to emergent group behaviors, including financial bubbles and recessions.

Park (2024) and Yang et al. (2025) both utilize multi-agent frameworks, but (Park, 2024) focuses on automating alert validation, whereas (Yang et al., 2025) simulates human behavior to model emergent market phenomena. Koa et al. (2024) takes a different approach by focusing on temporal relational reasoning, which emulates human-like temporal analysis. Therefore, the papers vary in their approach, from multi-agent systems to temporal reasoning.

The early detection of anomalies is a critical step in risk mitigation, allowing for proactive measures to be taken before significant market disruptions occur.

#### 2.6.3 Risk mitigation

Currently, there are no papers found that directly fit this category.

### 2.7 Financial content generation and data integration

This section covers research that utilizes LLMs for generating financial content or integrating large-scale datasets. The overarching aim is to synthesize information from diverse sources into coherent, actionable outputs that aid in decision-making and provide market insights. Key areas of focus include:

#### 2.7.1 Report generation/automation

- Nishida and Utsuro (2025) demonstrates automated production of financial news articles covering stock price fluctuations.
- Pop et al. (2024) discusses how LLMs streamline equity research reporting by automating significant portions of the writing process.

While both papers focus on automating financial content, Nishida and Utsuro (2025) is geared toward generating news articles with a focus on timeliness, whereas (Pop et al., 2024) focuses on the automation of more in-depth equity research reports.

#### 2.7.2 Financial LLM development and democratization

- Liu et al. (2023) introduces an open-source framework (FinGPT) designed to democratize financial LLMs. It covers data collection, fine-tuning, and various adaptation strategies, supporting a wide range of downstream applications.

### 2.8 Benchmarking, surveys, evaluation and datasets

This final category highlights studies that assess and benchmark the performance of LLMs in financial contexts. These works often involve surveys, comparative analyses, or the introduction of new evaluation frameworks—serving as foundational references for future research.

- Surveys and reviews: Several papers (Zhao et al., 2024; Nie et al., 2024; Kong et al., 2024; Ding et al., 2024; Kong et al., 2024) offer broad overviews of LLM applications, ranging from automating financial reports to deploying trading agents.
- Benchmarks: Papers (Li et al., 2024; Mateega et al., 2025; Krause, 2023) focus on the development of new benchmarks (e.g., InvestorBench, FinanceQA), enabling more systematic comparisons of LLM performance. Li et al. (2024) focuses on create a benchmark/standardized way to evaluate how well LLMs perform in financial analysis tasks.
- Comparisons: Abdelsamie and Wang (2024) benchmarks specialized financial LLMs, like Quantum, against human analysts and general-purpose LLMs to evaluate their market prediction accuracy and efficiency.
- Limitations: Papers (Bi et al., 2024; Chiang et al., 2025) delve into the practical potential and limitations of LLMs for financial forecasting and strategic decision-making, underscoring the need for ongoing evaluation in this rapidly evolving field.

## 3 LLM technical innovations, approaches

**Figure 2:**
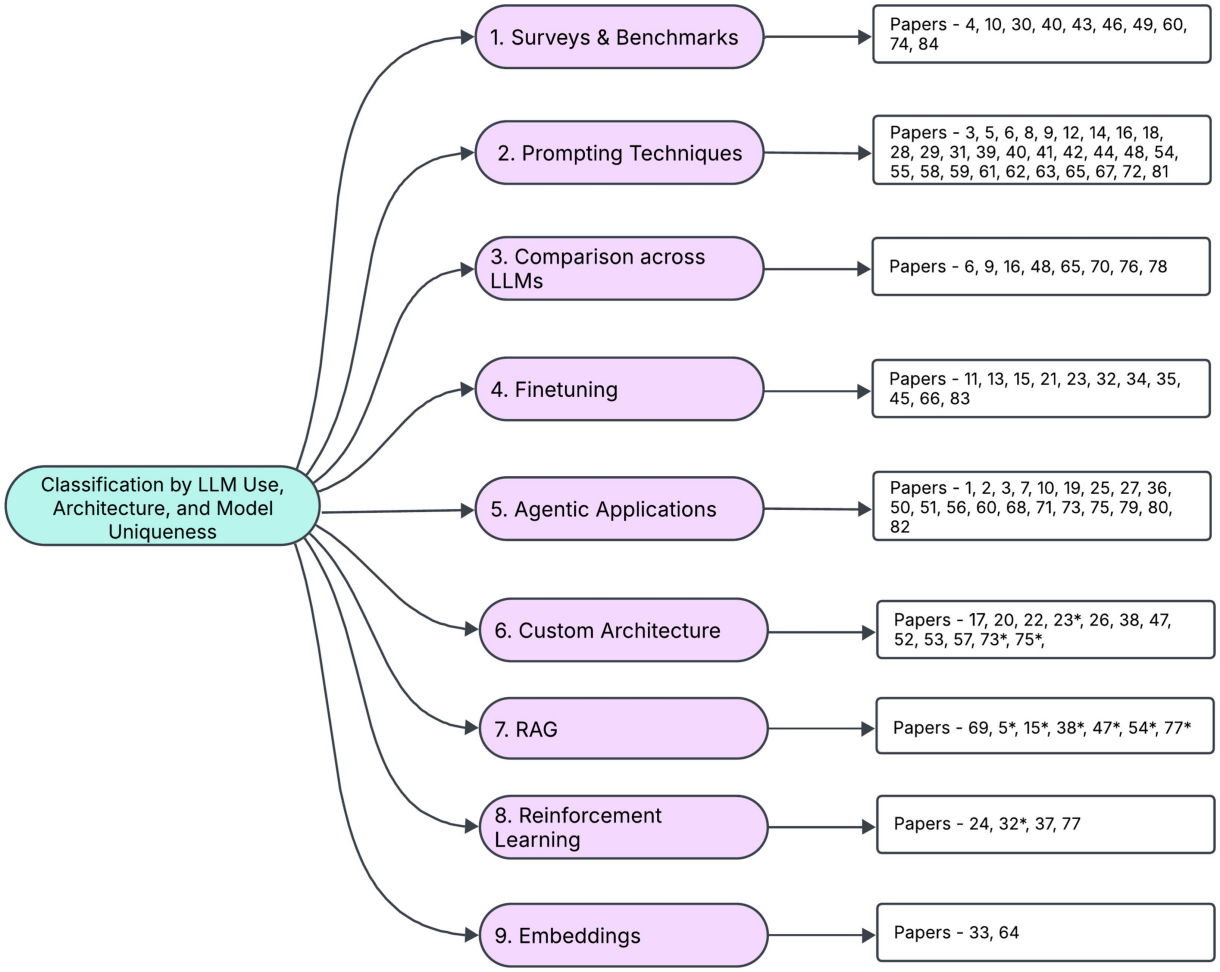
Categorization of research by LLM technique/methodology.

This section presents the key technical innovations underpinning recent research in the application of Large Language Models (LLMs) to Financial Stock Investing. We offer a comprehensive overview of the methodologies employed across these studies, highlighting the diverse techniques and nuanced approaches that drive progress in this field. Figure 2 provides a summary of the categorization of recent research by different approaches for LLMs.

### 3.1 Surveys and benchmarks

This category includes research that systematically evaluates the performance of LLM-based financial applications through benchmarks, surveys, and evaluation frameworks, providing critical insights into both the capabilities and limitations of current approaches.

#### 3.1.1 Broad overview

- Zhao et al. (2024) and Kong et al. (2024) provides a comprehensive overview of how LLMs are being applied in finance.
- Bi et al. (2024) surveys how AI—especially ChatGPT—can transform financial forecasting, addressing key challenges such as privacy and ethics.
- Kong et al. (2024) reviews the transformation of finance by LLMs, categorizing applications and presenting datasets, benchmarks, and methodologies for future research.

While all three papers provide broad overviews, Zhao et al. (2024) and Kong et al. (2024) offer a general view, Bi et al. (2024) focuses on the potential of a specific LLM, and Kong et al. (2024) provides a structured categorization of the field.

Note that the categorization is not completely mutually exclusive, some papers that fit in two categories are represented in the one category and by an asterisk in the next most relevant category.

#### 3.1.2 Application-specific overviews

- Sentiment analysis: Liu et al. (2024) provides a deep dive into the application of LLMs for financial sentiment analysis, using detailed datasets and case studies.
- Trading agents: Ding et al. (2024) reviews the landscape of LLM-based trading agents.

The application-specific overviews provide a foundation for more detailed technical evaluation.

#### 3.1.3 Technical overview

- Nie et al. (2024) offers an in-depth review of various approaches, detailing progress, prospects, and challenges while classifying applications into linguistic, sentiment, financial, and agent-based tasks.

#### 3.1.4 Benchmarking and bias evaluation

- Li et al. (2024) introduces InvestorBench, a standardized benchmark for evaluating LLM-based agents in financial decision-making across equities, cryptocurrencies, and ETFs, assessing their reasoning and decision-making capabilities using 13 different LLM backbones.
- Glasserman and Lin (2023) evaluates both “Look Ahead Bias” and the “distraction effect” by comparing sentiment-driven trading strategies based on original versus anonymized news headlines, using in-sample and out-of-sample tests to isolate and measure these biases.
- Yu (2023) presents a case study on US equity market news sentiment analysis revealing significant variability in LLM sentiment classification, highlighting inherent output volatility. This could act as a benchmark reference for future research.

Li et al. (2024) provides a broad benchmark for financial agents, while (Glasserman and Lin, 2023; Yu, 2023) focus on specific aspects of LLM performance, such as bias and output volatility. Glasserman and Lin (2023) and Yu (2023) both use real world data to evaluate LLMs, whereas (Li et al., 2024) builds a benchmark.

The findings from these benchmarking and bias evaluations inform the broader overviews and technical analyses.

### 3.2 Prompting techniques

Prompting techniques are pivotal in harnessing the capabilities of LLMs for financial applications, enabling precise control over model outputs and facilitating complex reasoning. This section explores various prompting methodologies, demonstrating their impact across diverse financial equity investing tasks, from forecasting to portfolio management.

#### 3.2.1 Zero-shot and few-shot prompting for financial analysis and forecasting

Paper 5: Uses GPT-4-32k with a diverse set of financial datasets (covering fundamental, market, and news data) and a Retrieval Augmented Generation (RAG)-like strategy to generate multi-horizon equity stock ratings. It employs both zero-shot and few-shot prompting for effective data integration.

- Nishida and Utsuro (2025) generates explanatory financial news articles about stock price movements by implementing few-shot learning and contrasting its performance with zero-shot methods.
- Pop et al. (2024) enhances stock trend prediction by applying a “denoising-then-voting” technique that combines few-shot learning with in-context prompt engineering.
- Mateega et al. (2025) automates the extraction of key elements from equity research reports by combining zero-shot/few-shot prompting with information retrieval.
- Chen et al. (2024) investigates ChatGPT’s forecasting tendencies by analyzing its extrapolation behavior, calibration, and inherent biases in predicting historical stock returns using a prompt-based approach.
- Lee et al. (n.d.) converts qualitative textual insights into quantitative market prediction scores by leveraging crafted prompts with dynamic few-shot examples.
- Swamy et al. (n.d.) extracts quantitative signals from qualitative data—such as moving averages and options volume—using innovative prompting strategies and integrates these signals with traditional quantitative features.
- Lefort et al. (2024) classifies financial news sentiment and de-noises aggregated outputs through zero-shot and few-shot prompting, supporting trading decisions on the NASDAQ.
- Chen et al. (2024) applies zero-shot classification to analyze over 77 million investor social-media posts, categorizing them into technical versus fundamental analysis and bullish versus bearish sentiment.
- Romanko et al. (2023) implements a two-step process where prompting (via zero-shot/few-shot methods) gathers stock suggestions, which are then refined using traditional portfolio optimization techniques (e.g., Markowitz mean–variance optimization).

These papers showcase the versatility of zero-shot and few-shot prompting, varying in their complexity and application. Papasotiriou et al. (2024) and Romanko et al. (2023) utilize RAG and optimization techniques, respectively, to enhance the prompting process, while other papers focus on direct application to sentiment analysis, forecasting, and data extraction.

The effectiveness of these prompting techniques extends to portfolio management, where tailored prompts can guide LLMs in generating and evaluating investment strategies.

#### 3.2.2 Prompting techniques in portfolio management

- Li et al. (2024) leverages zero-shot prompting to generate portfolios and assess asset diversification, comparing the performance of these portfolios against randomly selected ones.
- Ko and Lee (2024) uses ChatGPT in a zero-shot setting to produce asset class selections, which are quantitatively evaluated for diversity and performance.
- Fieberg et al. (2024) employs structured prompts across 32 different LLMs, each reflecting diverse investor profiles, to generate financial advice that is then evaluated for suitability, performance, and potential bias.
- Lu (2025) integrates a ChatGPT-based system—utilizing both zero-shot and few-shot prompting—into a robo-advisor framework to enhance investor understanding of alpha and beta, leading to improved portfolio choices.
- Bond et al. (2023) utilizes ChatGPT’s zero-shot NLP capabilities to analyze daily U.S. news summaries and generate a market sentiment indicator for short-term stock return forecasting.
- Wang et al. (2023) also covers the development of Alpha-GPT, an interactive system that uses prompt engineering and LLMs to generate creative trading signals, validated through alpha mining experiments.

These papers demonstrate the application of prompting for portfolio management, with variations in the use of zero-shot versus few-shot prompting, as well as the use of LLMs to generate investment advice, and trading signals. Fieberg et al. (2024) and Lu (2025) incorporate the analysis of investor profiles, and educational aspects, whereas the rest of the papers focus on pure portfolio creation and analysis.

#### 3.2.3 Chain of thought prompting

- Fatouros et al. (2024) uses GPT-4 as both a predictor and signal evaluator for stock selection by analyzing diverse financial data. It employs chain-of-thought and in-context learning techniques to enhance signal accuracy.
- Deng et al. (2022) adopts a semi-supervised approach by prompting an LLM with chain-of-thought techniques to generate weak sentiment labels from Reddit posts, which are then distilled into a smaller model using regression loss—achieving performance comparable to supervised methods.

These papers show how chain of thought prompting allows LLMs to create better outputs, by improving the reasoning process. Fatouros et al. (2024) uses it to improve signal accuracy, whereas (Deng et al., 2022) uses it to create better sentiment labels.

#### 3.2.4 Instruction prompting and context prompting

- Perlin et al. (n.d.) evaluates Google’s Gemini 1.5 Flash LLM for investment decision-making using anonymized U.S. market data. It queries the LLM with prompts that specify investment horizons and relevant financial inputs.
- Huang et al. (2024) implements scenario-based iterative prompt engineering to generate stock suggestions. These suggestions are subsequently screened using additional financial algorithms (NBESOA) for optimized portfolio construction.
- Lu et al. (2023) uses a prompt-based approach with data feeds from the Wall Street Journal and China State policy datasets to assess the efficacy of ChatGPT in providing financial stock investing recommendations.
- Dolphin et al. (2024) by combining LLM generative capabilities with advanced prompting and a validation framework using string similarity, the system extracts granular, per-company insights from news articles, demonstrating high accuracy and providing a live API and dataset for further research.

These papers showcase the use of instruction and context prompting across various applications, from evaluating LLM performance to generating stock suggestions and extracting market insights. Perlin et al. (n.d.) and Lu et al. (2023) focus on evaluation, whereas (Huang et al., 2024; Dolphin et al., 2024) focus on the creation of outputs.

#### 3.2.5 Knowledge generation prompting

- Cheng and Tang (2023) leverages GPT-4 to autonomously generate equity investment factors through knowledge inference without direct data input. It employs prompting strategies that guide GPT-4 in reasoning and generating these factors.

### 3.3 Comparison across LLM models

Comparative analysis of LLMs is crucial for understanding their relative strengths and weaknesses in financial equity applications. This section explores studies that benchmark different models across various tasks, highlighting the nuances in their performance and capabilities.

- Krause (2023) compares the performance of ChatGPT, Bard, and Bing on various financial tasks—including report/text generation, decision support, summarization, and general NLP—to assess their suitability for financial analysis.
- Wu (2024) evaluates and contrasts multiple LLMs (e.g., ChatGPT, Tongyi Qianwen, and Baichuan Intelligence) in generating news-based stock scores and predicting stock market returns.
- Shi and Hollifield (2024) assesses the predictive capabilities of GPT against traditional transformer models like BERT using economic data from the Federal Reserve Beige Book, to determine their effectiveness in financial market prediction.
- Tandon (2024) systematically reviews and compares various LLM techniques (including BERT and FinBERT) for stock market prediction by analyzing financial news headlines using historical datasets from Kaggle and Yahoo Finance.
- Voigt et al. (2024) adapts NLP-centric, Transformer-based architectures for stock price forecasting by leveraging the structural parallels between text sequences and time-series data, with the expectation that models excelling in language processing can capture temporal dependencies in financial markets.
- Abdelsamie and Wang (2024) Compares the performance of Quantum, an advanced LLM specialized for financial forecasting, with other general purpose LLMs—GPT-3, GPT-4, FinGPT, and FinBERT and human analysts. Employing a dataset of historical financial data, news headlines, and social media sentiment, the research systematically assesses predictive accuracy, response efficiency, and interpretability across models.
- Lopez-Lira and Tang (2023) compares the performance of various GPT models, BERT and Finbert to confirm accuracy and reliability in predicting stock prices based on new headlines while also suggesting an interpretability framework
- Kirtac and Germano (2024) covers a comparative analysis of LLMs, including GPT-3-based OPT model, BERT, and FinBERT, alongside traditional methods for financial news sentiment analysis, demonstrating that OPT significantly outperforms others in predicting stock market returns and generating substantial trading profits.

These papers vary in their scope and focus, from comparing general-purpose LLMs across diverse tasks (Krause, 2023; Wu, 2024) to evaluating specialized financial LLMs against human analysts and traditional models (Abdelsamie and Wang, 2024; Kirtac and Germano, 2024). Shi and Hollifield (2024), Tandon (2024) and Voigt et al. (2024) focus on comparing LLMs against traditional transformer models. Lopez-Lira and Tang (2023) adds the dimension of interpretability. The evaluations vary in the data used, and the metrics used.

The findings from these comparative studies inform the development of more effective prompting techniques and the selection of appropriate models for specific financial investing tasks.

### 3.4 Finetuning

Finetuning plays a critical role in adapting Large Language Models (LLMs) to the specific demands of financial stock investing tasks, enabling them to capture nuanced patterns and generate accurate predictions. This section reviews various finetuning methods used to adapt Large Language Models to financial tasks, ranging from instruction tuning to parameter-efficient finetuning and knowledge distillation.

#### 3.4.1 Finetuning/domain adaptation

- Guo and Hauptmann (2024) fine-tunes LLMs for stock return forecasting, integrating and comparing token-level embeddings from different LLM architectures.
- Valeyre and Aboura (2024) evaluates the Chronos model for time series prediction in financial markets by testing both its pre-trained and fine-tuned configurations using supervised forecasting.
- Aparicio et al. (2024) adapts BioBERT to the financial domain by fine-tuning it on curated financial textual databases, enabling sentiment analysis of press releases and financial texts around key biotech stock inflection points.
- Wang et al. (2024) this research developed InvestAlign, a method that constructs supervised fine-tuning training datasets for LLMs using theoretical solutions from simplified investment problems, rather than costly and privacy-sensitive real-user data, to better align LLM investment decisions with human investor behavior.

These papers demonstrate various approaches to domain adaptation, ranging from fine-tuning for specific prediction tasks (Guo and Hauptmann, 2024; Valeyre and Aboura, 2024) to adapting models from other domains (Aparicio et al., 2024) and creating synthetic datasets for finetuning (Wang et al., 2024). The data used, and the model architectures adapted vary significantly.

Instruction finetuning builds upon domain adaptation by incorporating specific instructions to guide the model’s learning.

#### 3.4.2 Instruction finetuning and instruction prompting

- Liang et al. (2024) constructs an instruction tuning dataset using a multi-step process—clustering company-related news to capture dissemination influence, enriching prompts with context and explicit instructions, and then fine-tuning the LLM—to enhance sentiment-based stock movement prediction.

#### 3.4.3 Parameter efficient finetuning (PEFT)

PEFT aims to reduce the computational and memory costs associated with finetuning large models

- Konstantinidis et al. (2024) implements LoRA fine-tuning on Llama 2 7B within a generator-classifier architecture for efficient financial sentiment analysis. [Custom Architecture]
- Liu et al. (2023) introduces FinGPT, an open-sourced framework that automates real-time financial data collection and adapts general-purpose LLMs for applications like robo-advising and sentiment analysis using LoRA/QLoRA fine-tuning, coupled with reinforcement learning (RLSP). [Reinforcement Learning]

Konstantinidis et al. (2024) and Liu et al. (2023) both utilize LoRA finetuning, but they differ in their architectures and applications. Konstantinidis et al. (2024) uses LoRA within a custom architecture, whereas (Liu et al., 2023) uses LoRA within its open-source framework, and also in conjunction with reinforcement learning.

#### 3.4.4 Combined instruction and parameter efficient finetuning

Combining instruction finetuning with parameter-efficient techniques allows for both task-specific adaptation and computational efficiency.

- Ni et al. (2024) employs a QLoRA-enhanced instruction fine-tuning strategy that combines base and external factors for improved stock prediction following earnings reports.
- Li et al. (2023) leverages both unsupervised and supervised LoRA on Llama2 alongside instruction fine-tuning on GPT3.5 to automate summarization and idea generation from diverse financial texts, thereby enhancing fundamental investment research.

#### 3.4.5 Instruction tuning with reinforcement learning

Reinforcement learning can be integrated with instruction finetuning to further refine model performance based on real-world feedback

- Zhao and Welsch (2024) includes finetuning of LLaMA 2 model with instruction tuning to incorporate human instructions. Additionally, reinforcement learning (RL) is used to incorporate stock market feedback by dynamically adjusting knowledge source weights within a RAG module, improving financial sentiment analysis and stock price movement prediction.

Finetuning can also be used to enhance embedding generation for predictive modeling.

#### 3.4.6 Fine tuning for embedding generation and predictive modeling

- Chen et al. (2022) utilizes LLMs (e.g., ChatGPT and LLaMA) to extract contextualized representations from news text for predicting expected stock returns. The study demonstrates that LLM-based predictions, which capture broader article context and complex narratives, significantly outperform traditional technical signals across multiple global equity markets.

#### 3.4.7 Finetuning and knowledge transfer

- Das et al. (2024) combines financial textual data (news and sentiment) with price signals by leveraging large pretrained models (e.g., LLaMA-2-13B, Mistral, Gemma). These models are further fine-tuned or prompted in a zero−/few-shot manner to generate automated trading actions, facilitating effective knowledge transfer.

### 3.5 Agentic frameworks

Agent-based systems have the potential to transform financial stock and equity investing by automating complex decision-making processes. This section reviews various agentic applications, from single-agent solutions to sophisticated multi-agent frameworks, highlighting their diverse approaches and impacts. Research on agent-based frameworks in financial stock and equity investing is still emerging, with only 20 of the 84 reviewed papers focusing on agent systems. (Note: Ding et al. (2024) is a survey and is covered in Section 3.1.1.).

#### 3.5.1 Single-agent applications

- Yue and Au (2023) GPTQuant uses prompt templates and LangChain to create a conversational AI agent that generates Python code for investment research, streamlining the analysis process.
- Koa et al. (2024) covers the development of a Summarize-Explain-Predict (SEP) framework, which utilizes a self-reflective agent and Proximal Policy Optimization (PPO) to train an LLM to autonomously generate explainable stock predictions, achieving superior performance in both prediction accuracy and portfolio construction.

#### 3.5.2 Multi-agent systems using chain-of-thought (CoT)

Multi-agent systems expand on these single-agent capabilities by coordinating multiple agents to perform complex tasks

- Zhou et al. (2024) utilizes a multi-agent CoT system to automate equity research and valuation by combining quantitative and qualitative analysis. It dynamically updates its data using three specialized agents (Data CoT, Concept CoT, and Thesis CoT) that emulate human analyst reasoning, resulting in high-quality, timely research.
- Wang et al. (2024) to understand LLM’s reasoning approach for trading decision, study introduce FS-ReasoningAgent, a multi-agent framework that separates reasoning into factual and subjective components, demonstrating enhanced LLM trading performance and showing that subjective news drives returns in bull markets, while factual data performs better in bear markets.

Zhou et al. (2024) and Wang et al. (2024) both employ multi-agent CoT systems, but they focus on different aspects of financial investing. Zhou et al. (2024) automates equity research, while (Wang et al., 2024) analyzes the impact of factual versus subjective news on trading decisions.

#### 3.5.3 Multi-agent systems with coordinated networks

Coordinated networks further enhance multi-agent systems by enabling complex interactions and adaptive decision-making.

- Kou et al. (2024) proposes a multi-step framework where LLMs extract alpha factors from multimodal financial data. These factors are integrated into a multi-agent system with dynamic weight-gating to produce an adaptive composite alpha formula for enhanced portfolio management.
- Zhang et al. (2024) introduces a multi-agent system, “StockAgent,” which simulates investor trading behavior in a realistic market environment. Agents make trading decisions based on various external factors.
- Park (2024) presents a multi-agent framework for anomaly detection in financial markets. It employs specialized agents for data conversion, expert web analysis, institutional knowledge application, cross-checking, and report consolidation, thereby automating anomaly alert validation in the S&P 500, reducing manual verification.
- Xiao et al. (2024) deploys a multi-agent framework (TradingAgents) that simulates a trading firm by assigning roles such as fundamental, sentiment, and technical analysts, along with risk management agents. These agents collaboratively debate and synthesize diverse analyses and historical data to inform trading decisions, outperforming baseline models on key metrics
- Fatemi and Hu (2024) develops a multi-agent system (FinVision) that analyzes diverse multimodal financial data (including text and charts) using a “reflection module” that reviews past trading signals—especially visual cues—to improve stock market predictions.
- Xing (2024) proposes a heterogeneous LLM agent framework for financial sentiment analysis, where specialized agents collaboratively discuss identified error types to improve accuracy without additional fine-tuning.
- Yang et al. (2024) presents an open-source multi-agent platform (FinRobot) for financial tasks that employs a layered architecture. It leverages Financial Chain-of-Thought to decompose complex problems, dynamically selects LLM strategies, and integrates diverse models through LLMOps and DataOps.
- Yang et al. (2025) presents TwinMarket (a multi agent framework) that simulates individual behaviors and interactions to demonstrate how they lead to collective dynamics and emergent phenomena, such as financial bubbles and recessions, within a simulated stock market.
- Li et al. (2023) TradingGPT (single and multi-agent system) utilizes three memory layers with custom decay mechanisms, inter-agent debate, and individualized trading traits to enable agents to effectively integrate historical data and real-time market signals for enhanced trading decisions. Note the layered memory also fits in custom architecture

These papers showcase diverse approaches to multi-agent systems with coordinated networks, ranging from alpha factor extraction (Kou et al., 2024) to anomaly detection (Park, 2024) and trading simulation (Zhang et al., 2024; Xiao et al., 2024; Fatemi and Hu, 2024; Yang et al., 2025; Li et al., 2023). The complexity of the systems, and the tasks performed vary significantly. Xing (2024) and Yang et al. (2024) focus on frameworks, whereas the rest focus on applications.

#### 3.5.4 Multi-agent systems with reinforcement learning

Reinforcement learning can further enhance multi-agent systems by enabling agents to learn from experience and adapt to changing market conditions.

- Yu et al. (2024) introduces FinCon, a hierarchical multi-agent system modeled after real-world investment firms. It employs a “conceptual verbal reinforcement” mechanism where agents self-critique and update their investment beliefs to guide future actions, thereby improving performance and reducing unnecessary communication.
- Yu et al. (2023) presents FinMem a multi-agent architecture that incorporates three core modules: Profiling for agent customization, a layered Memory module that emulates human trader cognition for efficient hierarchical data assimilation, and Decision-making to translate insights into investment actions; this design allows the agent to self-evolve, adapt to market cues, and surpass human perceptual limits, resulting in improved trading performance.
- Li et al. (2024) CryptoTrade, incorporates a reflective mechanism to analyze prior trading outcomes and refine daily decisions, demonstrating superior performance compared to traditional strategies and time-series baselines across various cryptocurrencies and market conditions.

These papers demonstrate the use of reinforcement learning in multi-agent systems, with variations in the reinforcement mechanisms and applications. Yu et al. (2024) uses a conceptual verbal reinforcement mechanism, whereas (Yu et al., 2023) focuses on a multi module architecture, and (Li et al., 2024) uses a reflective mechanism.

### 3.6 Custom architecture

Custom architectures are pivotal in pushing the boundaries of LLM applications in finance, enabling the development of specialized models that address unique challenges. This section explores a variety of innovative architectural approaches, from knowledge distillation to hybrid and multi-modal frameworks. Please note multi-agent architectures are covered explicitly in the section 3.5—Agentic Applications.

#### 3.6.1 Knowledge distillations

Knowledge distillation includes the transfer knowledge from a large “teacher” model to a smaller “student” model.

- Bhat (n.d.) employs a computationally efficient distilled LLM to extract emotional tone and intensity from financial news headlines. The distilled model’s outputs are fed into classification algorithms for predicting stock price direction, demonstrating that emotion analysis alone can rival the performance of traditional financial data methods.

#### 3.6.2 Transfer learning and foundation models

Transfer learning and foundation models extend this concept by leveraging pre-trained knowledge for broader applications. Guo and Shum (2024) demonstrates the power of transfer learning and foundation models in capturing universal market patterns.

- Guo and Shum (2024) utilizes a novel LLM structure designed for large-scale investment applications. The model, termed Large Investment Model (LIM), employs a “foundation model” approach by training on vast financial datasets to learn universal market patterns, which are then transferred via transfer learning to develop specialized and efficient investment strategies for various financial tasks.

#### 3.6.3 Retrieval-augmented and agent-based architectures

Retrieval-augmented and agent-based architectures further enhance LLM capabilities by integrating external knowledge and dynamic interactions.

- Fatouros et al. (2025) introduces MarketSenseAI 2.0, which combines Retrieval-Augmented Generation (RAG) with LLM agents for comprehensive stock analysis and selection. This framework processes diverse data types—including financial news, historical prices, company fundamentals, and macroeconomic indicators—with RAG handling SEC filings, earnings calls, and institutional reports. Empirical results on S&P 100 stocks (2023–2024) indicate cumulative returns of 125.9% versus an index return of 73.5%.
- Kim and Oh (n.d.) presents an integrated system that fuses RAG with LangChain to dynamically retrieve and synthesize external financial data, producing real-time, contextually enriched stock analysis reports.

Fatouros et al. (2025) and Kim and Oh (n.d.) both use RAG, but (Fatouros et al., 2025) uses RAG in combination with LLM agents.

#### 3.6.4 Time series and temporal reasoning architectures

Time series and temporal reasoning architectures complement other forms by focusing on the temporal dynamics of financial data.

- Wang et al. (2024) proposes StockTime, a specialized LLM architecture designed explicitly for stock price time series data. By treating stock prices as consecutive tokens, StockTime extracts textual information (e.g., correlations, trends, timestamps) and integrates this with time series data into an embedding space. This multimodal fusion yields more accurate predictions while reducing memory usage and runtime costs.
- Koa et al. (2024) introduces Temporal Relational Reasoning (TRR), a framework that combines LLM-based zero-shot text interpretation with cognitively inspired components (memory, attention, reasoning) to track and aggregate news impacts over time. This structured approach improves detection of impending portfolio crashes by modeling temporal relationships among events and stocks.

Wang et al. (2024) and Koa et al. (2024) both address temporal reasoning, but they differ in their approaches. Wang et al. (2024) focuses on treating time series data as tokens, while (Koa et al., 2024) integrates cognitive components.

#### 3.6.5 Hybrid and multi-modal frameworks

Hybrid and multi-modal frameworks integrate diverse data types and models to capture complex market relationships.

- Di et al. (2024) proposes a custom architecture for securities index prediction that integrates LLM-driven knowledge extraction with a heterogeneous graph and a Graph Neural Network (GNN) to capture complex market relationships.
- Tong et al. (2024) presents a novel, integrated two-part framework (Ploutos) that combines specialized experts for multi-modal data analysis with tailored prompting and dynamic token weighting to enhance interpretability in stock movement prediction.
- Ding et al. (2023) introduces a novel framework that combines a Local–Global model for integrating stock features with LLM-derived semantic information and employs self-correlated reinforcement learning to align these embeddings within a shared semantic space.

These papers showcase diverse hybrid and multi-modal frameworks, combining various techniques to capture complex market relationships. Di et al. (2024), Tong et al. (2024) and Ding et al. (2023) all use different methods to combine different data types and models.

### 3.7 Retrieval-augmented generation (RAG)

Retrieval-Augmented Generation (RAG) in stock investing enables LLMs to provide more accurate and contextually relevant financial insights by dynamically retrieving and incorporating up-to-date information from diverse sources like news articles, financial reports, and market data.

We observe that only a few papers out of the 84 research papers incorporate RAG in some form, with just two using it as the primary technique.

- Li et al. (2024) introduces AlphaFin, a data set combining traditional research datasets, real-time financial data, and handwritten chain-of-thought (CoT) data to address the limitation of limited financial training dataset availability. Further they introduce a RAG based, two phased framework (StockChain) for financial analysis.

Several papers incorporate RAG with diverse techniques. Papers (Papasotiriou et al., 2024; Romanko et al., 2023) combine RAG with zero-shot and few-shot prompting; Papers (Kim and Oh, n.d.; Fatouros et al., 2025) utilize RAG with agents and (Zhao, 2024) integrates RAG with reinforcement learning while (Zhao and Welsch, 2024) uses RAG in combination with reinforcement learning and instruction tuning.

### 3.8 Reinforcement learning

This section examines the integration of Reinforcement Learning (RL) with Large Language Models (LLMs) to advance financial trading. Recent studies demonstrate RL’s efficacy in enabling LLM-driven adaptive strategies, particularly in achieving regime-adaptive execution, enhancing the explainability of trading decisions, and improving portfolio performance across volatile market conditions.

#### 3.8.1 Regime adaptation via reinforcement learning

- Saqur and Rudzicz (2024) Leverages Reinforcement Learning from Market Feedback (RLMF), a regime-adaptive market execution method, to dynamically adjust LLM behavior in real-time. This model-agnostic approach, demonstrated with Llama-2 7B, utilizes intrinsic market rewards and a teacher-student dual-phase pipeline (iterative train and execute cycles) to improve predictive accuracy by 15% over models like GPT-4o, effectively circumventing the need for human-labeled data. This highlights a trend in utilizing RL to reduce reliance on extensive human-labeled datasets.

#### 3.8.2 Adaptive and explainable trading systems

- Gu et al. (2024) presents a framework that fuses LLMs with Reinforcement Learning for margin trading. The LLM system analyzes diverse financial data to produce market forecasts with explainable reasoning, which are then integrated with RL to dynamically adjust trading positions, significantly boosting returns and Sharpe ratios while enhancing transparency in portfolio management.
- Zhao (2024) introduces the Hierarchical Reinforced Trader (HRT), an adaptive Retrieval-Augmented Generation (RAG) framework for LLMs, employing bi-level Deep Reinforcement Learning (DRL) and an enhanced Univariate Flagging Algorithm (UFA) for model interpretability. This framework demonstrates significant improvements in portfolio performance and risk management across diverse market conditions. Portfolio comparisons to the S&P 500 reveal favorable results in both bull and bear/volatile market scenarios.

### 3.9 Embedding based methods

Embedding-based methods leverage the semantic understanding capabilities of Large Language Models (LLMs) to transform textual financial data into dense vector representations. This section explores recent applications of LLM embeddings in financial analysis, focusing on their use in stock prediction and communication evaluation.

- Chen et al. (2022) utilizes state-of-the-art LLMs (e.g., ChatGPT, LLaMA) to extract contextualized embeddings from news articles. These embeddings capture nuanced language features—such as negation and complex narratives—and are used as input features to predict stock returns, outperforming traditional technical signals and simpler NLP methods across diverse global equity markets.
- Chiang et al. (2025) leverages LLM-based vector embeddings derived from 192,000 earnings call transcripts to quantify the semantic alignment and relevance of Q&A segments, providing a novel metric for evaluating investor communication effectiveness. This method allows for a deeper understanding of the relationship between the questions asked, and the answers given, in earnings calls.

## 4 Data sets and models

**Figure 3:**
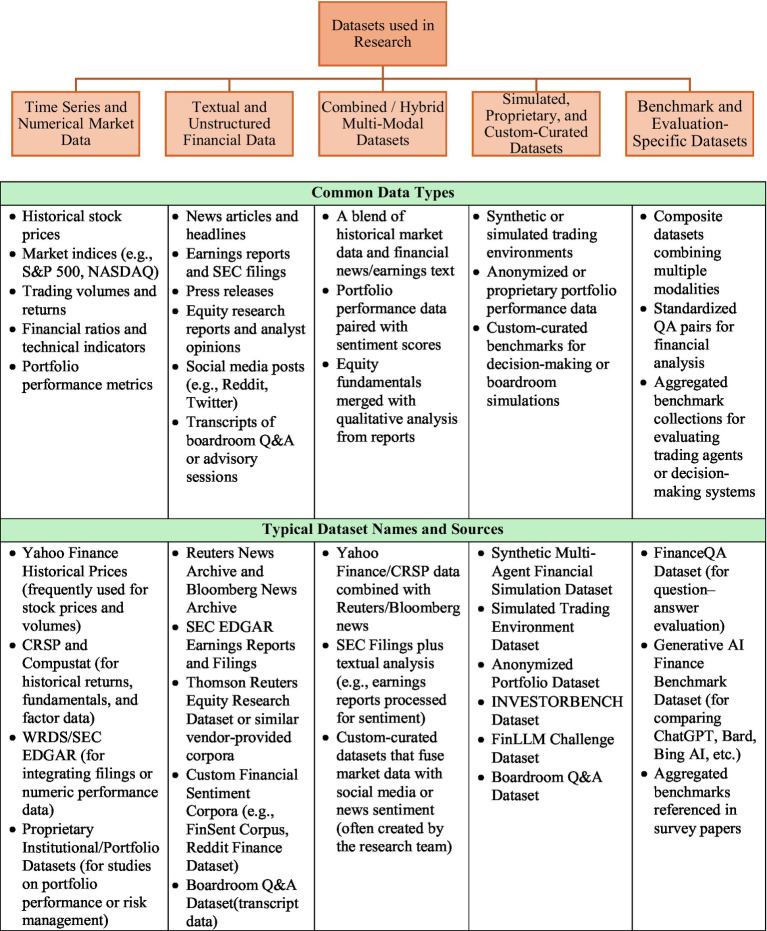
Datasets used across 84 different research papers.

For a comprehensive view on data sets used across the 84 different research is presented Figure 3.

Majority of the datasets used in the 84 research studies are public data sets available through SEC, Edgar, Yahoo Finance, News, Company websites, Social media. Only a few limited studies use specialized datasets—Simulated Trading Environment Dataset (Zhang et al., 2024; Xiao et al., 2024), Anonymized Portfolio Dataset (Perlin et al., n.d.), INVESTORBENCH Dataset (Li et al., 2024), FinLLM Challenge Dataset (Das et al., 2024), Boardroom Q&A Dataset (Chiang et al., 2025), FinanceQA Dataset (for question–answer evaluation) (Mateega et al., 2025).

**Figure 4:**
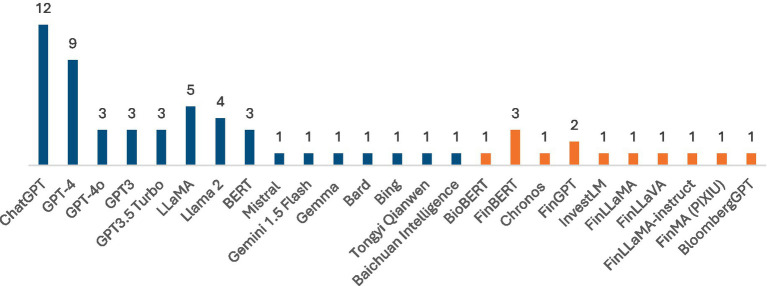
General purpose (blue) and fine-tuned (orange) LLMs used in research.

A central theme across the 84 reviewed papers is the diversity of LLMs employed for financial tasks. Some studies leverage generic transformer models (e.g., GPT, LLaMA) with minimal modifications, while others adopt domain-specific architectures fine-tuned on large corpora of financial text. Figure 4 provides the distribution of LLMs used in research so far. Out of the 84 research papers evaluated, 50 papers provide specific reference to LLM names with a majority around 49 papers mentioning the use of general purpose LLMs such as GPT, Llama, BERT, others. A few studies present domain specific LLMs. Table 1 provides an overview of financial LLMs, including details on base models, parameters and key focus areas.

**Table 1:** Overview of financial LLMs for equity investing.

Model name	Base model	Parameters	Specialization	Key features
BloombergGPT	BLOOM	~50B	Financial text (news, reports, press)	Trained on combined general + financial corpus; strong performance on news-based tasks
FinGPT	Various	Varies	Financial datasets	Fine-tuned with Low-Rank Adaptation (LoRA); dissemination-aware and context-enriched
InvestLM	LLaMA-65B	65B	Financial investment tasks	Fine-tuned with a curated dataset for investment-related applications; focuses on advanced RL integration
FinLLaMA	LLaMA-2	52B tokens	Financial sentiment classification, trading simulations	Pre-trained on financial corpus; instruction fine-tuned with 573 K financial instructions
FinLlama	LLaMA-2 (7B)	7B	Sentiment analysis for algorithmic trading	Fine-tuned for sentiment valence and strength classification; optimized using LoRA
FinLLaVA	FinLLaMA	N/A	Multimodal financial data (text, tables, charts)	Trained with 1.43 M image-text instructions to handle complex financial data types
FinMA (PIXIU)	LLaMA (7B & 70B)	7B, 70B	Financial sentiment analysis and NLP tasks	Fine-tuned on financial datasets
FinBERT	BERT	N/A	Financial NLP tasks	Pre-trained on general and financial corpora; uses multi-task learning
FLANG	Custom	N/A	Financial corpus	Domain-specific model

Overall, the proliferation of finance-specific LLMs underscores a broader industry trend toward domain adaptation, multi-agent frameworks, and hybrid modeling approaches that harness the unique strengths of LLMs and insights from financial data.

## 5 Discussion

### 5.1 Strengths of existing research

The reviewed literature highlights several strengths in using LLMs for stock investing:

- **Comprehensive Data Integration:** Many studies successfully integrate structured (e.g., financial statements, historical prices) and unstructured (e.g., news articles, earnings call transcripts, social media) data to enhance predictive accuracy.
- **Breadth of Coverage (Application & LLM Techniques):** Research on use of LLMs in stock investing so far covers a broad set of financial end-use case applications (sentiment analysis, equity research, stock prediction, portfolio management, algorithmic trading, others) and diverse set of LLM techniques ranging from simple prompt based methods, to fine tuning, the use of LLM agents for automated workflows and decisions and the proposals of novel custom architectures.
- **Usage of General Purpose and Domain Specific LLM Models:** Majority of the research studies conducted so far use general purpose LLMs such as GPT 3, 4, LlaMA, BERT and others. Only a few studies use or present fine-tuned versions of general purpose LLMs such as (FinGPT, FinLlama, BioFinBERT) for improved performance on financial investing tasks.
- **Advancements in Model Architectures:** Several studies introduce novel frameworks such as Ploutos (for integrating numerical and textual data) and using a combination of generator and classifier model, StockTime (for time-series adaptation), and MarketSenseAI (for multi-modal analysis using RAG). These innovations improve LLM adaptability to financial markets.
- **Validation of potential to disrupt Sentiment Analysis and Investment Research:** Several research studies demonstrated superior performance of LLMs when used in sentiment analysis to capture signals from large data sets (news, analyst reports and social media) compared to traditional methods. Similarly, research confirmed LLMs streamlining equity research (e.g., GPTQuant for report generation, FinRobot for sell-side research automation) and support algorithmic trading through real-time processing and decision-making.
- **Emerging Multi-Agent and Reinforcement Learning Systems:** Research in AI-driven trading strategies has progressed beyond rule-based models to reinforcement learning and multi-agent frameworks that dynamically adjust to market conditions, improving adaptability and efficacy of predictions, risk management.
- **Benchmarking and Standardization:** Studies such as InvestorBench and FinanceQA offer benchmarking frameworks to systematically evaluate LLM performance in finance, providing a foundation for more structured comparisons.
- **Research Delivery in Constrained environments:** Access to computational resources—such as GPUs for model fine-tuning could be a limitation. Similarly, lack of transparency and documentation on closed source models is another limitation for experimentation and Model interpretability. However, the breadth of research so far despite these limitations is notable.

### 5.2 Limitations of existing research studies

Despite these strengths, several limitations remain:

- **Limited Real-World Testing:** While many studies demonstrate promising results in controlled environments, few have tested LLM-driven investment strategies in real-world trading conditions. Challenges such as execution slippage, transaction costs, and market impact are often overlooked.
- **Data Quality and Bias:** LLMs are highly sensitive to the quality of the training data and the data used for inference. Many LLM-driven models depend on real-time market data and unstructured text sources, making them vulnerable to biased, manipulated, or misleading information, leading to potential overfitting and poor generalization. There is a potential risk for bad actors and attackers to manipulate data to distort investing outcomes and money flow. Out of the 84 research papers, only few research studies cover the data quality limitations and some factor bias considerations.
- **Interpretability and Explainability Challenges**: The black-box nature of many LLM-based models limits transparency, making it difficult for investors and regulators to understand, trust, and justify AI-driven investment decisions. This lack of interpretability hinders broader adoption and raises regulatory concerns in financial markets. GPT based models used in many research paper so far are closed-source and do not provide visibility on the model specifications, limiting explainability.
- **Context window Limitations:** All existing research studies focus on data sets with limited sizes and scope. None of the studies have elaborated or found a scalable solution for the high context window of inputs required for stock investing use cases- examples include PDF files with 100 + pages. Evaluating and
- **Challenges in Back-Testing and Validation:** The risk of data leakage in back-testing remains a critical concern. Most studies fail to rigorously test against out-of-sample data or consider survivorship bias, which can inflate performance metrics.—mention few studies that account for this
- **Regional Coverage:** Most research studies are focused on the US and China markets with one study covering data from the Japan market. Scalability of the findings and frameworks to data from markets in other regions remains an open question.
- **Coverage across Investment types:** Majority of the research conducted so far focusses on broader stock investing in the construct of long term/value investing. Research focus on day trading use cases or High frequency trading is unexplored.
- **Limited Exploration of Non-Equity Asset Classes:** Most research focuses on stock investing, with limited exploration of LLM applications in commodities, fixed income, or options markets, which require different risk assessment models.

By addressing these gaps, future research can refine LLM applications in financial investing, making them more accurate, scalable, and aligned with industry needs.

### 5.3 Research gaps and future directions

To overcome current limitations and further enhance the utility of LLMs in stock investing, future research should focus on:

- **Hybrid Modeling Approaches:** Integrating LLMs with traditional quantitative and AI models (e.g., econometric, factor models) and can leverage the strengths of both methodologies, could improve predictive performance and decision-making reliability.
- **Reasoning Models**: Most general purpose and generic LLMs used in the research so far are derived from GPT, BERT and Llama. There is limited to none reference to usage of reasoning models such as GPT o1, GPT o1mini, Deep Seek R1, others.
- **Efficiency Improvements- Solving for Computational Overhead and Latency:** The large size and complexity of LLMs often result in high computational costs and latency issues, making real-time trading applications challenging. Advancements in model optimization—such as distillation, quantization, and efficient fine-tuning techniques like LoRA—could reduce computational overhead and latency, making real-time applications more feasible. There is a huge potential for model architectural and algorithmic advances to meet unique needs of stock investing use cases.
- **Scalability of Multi-Agent AI Systems:** While multi-agent LLM frameworks have shown initial promise in controlled setups, their scalability, coordination mechanisms and reliability in high-stakes financial environments remain an open challenge.
- **Enhanced Explainability and Interpretability:** Developing new interpretability frameworks tailored to LLMs in financial/stock investing applications will be crucial for building stakeholder trust and ensuring regulatory compliance. Research into techniques that demystify LLM outputs is needed. Future work must address the ethical implications of automated decision-making
- **Ethical & Regulatory considerations:** The ethical and regulatory implications of deploying LLMs in equity markets require more comprehensive investigation to ensure responsible adoption and compliance with financial regulations. Current research, while acknowledging issues such as data biases and model interpretability, often overlooks the broader ethical concerns, including the potential for LLMs to amplify market manipulations through biased or misleading data inputs, such as orchestrated social media campaigns or falsified financial reports. Additionally, the lack of transparency in closed-source models (e.g., GPT-based systems) raises concerns about accountability, particularly when these models influence high-stakes investment decisions. Regulatory frameworks, such as those enforced by the SEC or ESMA, demand rigorous validation and explainability of AI-driven strategies, yet few studies address compliance with these standards or the ethical risks of over-reliance on automated systems. Future research should prioritize developing frameworks for ethical AI governance, including robust auditing mechanisms to detect and mitigate biases, transparent reporting protocols for LLM-driven decisions, and alignment with global financial regulations to foster trust and ensure equitable market participation.
- **Solving for Large Context Window:** To ensure true scalability of use in financial applications, the context window limitations of LLMs will need to be solved. Research on this topic will be vital for true scalability.
- **Maintenance and Domain Adaptation:** Domain-specific LLMs require continuous fine-tuning and updates to remain effective as market conditions evolve. This can be resource-intensive and may limit scalability.
- **Addressing Bias and Data Manipulation Risks:** Future research should develop more robust techniques to detect and mitigate biases in financial datasets, including adversarial attacks on AI-generated trading signals.
- **Adaptive Learning and Market Feedback Integration:** Reinforcement learning from real market interactions should be further explored to enable self-improving models that adapt dynamically to changing market conditions
- **Expansion to Broader Financial Instruments:** While most studies concentrate on equities, LLM applications should be extended to alternative asset classes, such as bonds, derivatives, and crypto markets, to assess their predictive power across different financial products.
- **Cross-Regional and Cross-Market Evaluations:** LLM-based investment models should be tested across different geographic markets and economic conditions to assess their generalizability and robustness.
